# Eco-evolutionary dynamics, density-dependent dispersal and collective behaviour: implications for salmon metapopulation robustness

**DOI:** 10.1098/rstb.2017.0018

**Published:** 2018-03-26

**Authors:** Justin D. Yeakel, Jean P. Gibert, Thilo Gross, Peter A. H. Westley, Jonathan W. Moore

**Affiliations:** 1 School of Natural Sciences, University of California, Merced, CA 95340, USA; 2 The Santa Fe Institute, Santa Fe, NM 87501, USA; 3 Department of Engineering Mathematics, University of Bristol, Bristol BS8 1TH, UK; 4 Department of Fisheries, University of Alaska Fairbanks, Fairbanks, AK 99775, USA; 5 Earth_2_Oceans Research Group, Simon Fraser University, Burnaby BC, Canada V5A 1S6

**Keywords:** salmon metapopulations, straying, dispersal, eco-evolutionary dynamics, alternative stable states

## Abstract

The spatial dispersal of individuals plays an important role in the dynamics of populations, and is central to metapopulation theory. Dispersal provides connections within metapopulations, promoting demographic and evolutionary rescue, but may also introduce maladapted individuals, potentially lowering the fitness of recipient populations through introgression of heritable traits. To explore this dual nature of dispersal, we modify a well-established eco-evolutionary model of two locally adapted populations and their associated mean trait values, to examine recruiting salmon populations that are connected by density-dependent dispersal, consistent with collective migratory behaviour that promotes navigation. When the strength of collective behaviour is weak such that straying is effectively constant, we show that a low level of straying is associated with the highest gains in metapopulation robustness and that high straying serves to erode robustness. Moreover, we find that as the strength of collective behaviour increases, metapopulation robustness is enhanced, but this relationship depends on the rate at which individuals stray. Specifically, strong collective behaviour increases the presence of hidden low-density basins of attraction, which may serve to trap disturbed populations, and this is exacerbated by increased habitat heterogeneity. Taken as a whole, our findings suggest that density-dependent straying and collective migratory behaviour may help metapopulations, such as in salmon, thrive in dynamic landscapes. Given the pervasive eco-evolutionary impacts of dispersal on metapopulations, these findings have important ramifications for the conservation of salmon metapopulations facing both natural and anthropogenic contemporary disturbances.

This article is part of the theme issue ‘Collective movement ecology’.

## 1. Introduction

Intraspecific diversity can increase the resilience and stability of species or metapopulations [1]. This diversity–stability linkage can arise when there are asynchronous population dynamics within the metapopulation. Such asynchrony will increase the potential for demographic rescue [2,3] and also decrease the variability of processes that integrate across the metapopulation [4]. For example, different responses to climate variability within populations of a rare plant reduced fluctuations in abundance [5]. This statistical buffer has traditionally been quantified as the portfolio effect (PE), which is the ratio of the population's coefficient of variation (CV) to the CV of the aggregated metapopulation [6]. Larger PEs are expected to increase the robustness of metapopulations to external disturbances, and by extension promote persistence [6]. By contrast, homogenization of populations leading to greater synchronization and weakened PE may be a harbinger of metapopulation collapse and extinction [7].

Permanent movement of individuals among local populations (i.e. dispersal) can have a large influence on metapopulation persistence [8]. Dispersal facilitates evolutionary rescue, whereby immigration of individuals with heritable adaptive traits can rescue small populations from local extinction in the context of maladaptive environmental change [9,10]. Dispersal also enables demographic rescue, when depressed or extirpated populations are recolonized by immigrants from the rest of the metapopulation. On the other hand, high rates of dispersal may synchronize the dynamics of populations and subsequently increase the risk of extinction of the entire metapopulation [3,11]. Dispersal may also introduce maladapted individuals into habitats that are host to different environmental conditions, possibly lowering the mean fitness of the recipient population [12,13]. More broadly, dispersal can provide a mechanism by which phenotypes are sorted in space rather than time and facilitates the spread of potentially maladaptive genes [14]. Dispersal in this case may lead to genetic homogenization that erodes the asynchrony underpinning PEs and metapopulation persistence.

There is growing appreciation that a combination of abiotic, biotic and anthropogenic factors can control the rate of dispersal among populations [15–20]. Migratory populations that return to breeding sites for reproduction can be linked to each other by some proportion of the population that disperses into the ‘wrong’ site. Recently, the role of social interactions to lead to collective navigation has been hypothesized as a mechanism shaping the success of philopatric migrations [21–23]. The collective navigation hypothesis posits that the rate at which individuals disperse may be linked to individual-level error, which is diminished by migrating in groups and pooling individual choices [21,23,24]. Thus, dispersal rates can be higher at lower population abundances [25], which can in turn profoundly influence the eco-evolutionary dynamics of metapopulations.

The eco-evolutionary impacts of dispersal probably have implications for conservation and management in key taxa such as in migratory salmon [26–28]. While anadromous salmonid fishes (genera *Oncorhynchus* and *Salmo*) are renowned for returning to their natal spawning habitats with high accuracy and precision after years at sea [17,29,30], some individuals disperse (termed ‘straying’ and used synonymously with dispersal hereafter) to non-natal sites to spawn [31,32]. Straying provides a mechanism for the colonization of new or connected habitats following glacial retreat or large-scale geomorphic landscape change [32]. Salmon appear to operate as metapopulations, where populations are in part reproductively isolated in discrete habitat patches, but linked by some level of straying [33,34]. Although extensive work has been done to document the extent of straying from donor populations into recipient populations [17,18], only recently have the abiotic, biotic and anthropogenic influences of straying behaviours been investigated systemically [35–37]. Straying among salmon may be influenced by environmental factors such as water temperature, human activities such as hatchery practices and population density, as predicted by the collective navigation hypothesis [23,38]. Straying can introduce new maladaptive genotypes into the recipient population, while the ensuing genetic homogenization could synchronize population dynamics and erode PEs [7,39,40]. Given that locally distinct populations are often linked by straying, there is an opportunity and need to understand the fundamental and applied consequences of straying for metapopulation persistence, conservation and management.

Here, we seek to explore how collective density-dependent straying influences the stability and robustness of metapopulations through ecological and evolutionary processes. To address this question, we build upon an established eco-evolutionary model of two populations occupying different sites that are linked by straying individuals, each with an associated trait distribution subject to natural selection determined by local conditions [12]. Specifically, we compared (a) density-independent (constant) straying with (b) density-dependent straying as a function of the rate at which individuals stray and the strength of collective behaviour across (c) increasing environmental heterogeneity, by assessing two measures of metapopulation robustness: the PE and the time required for recovery following an induced disturbance. This model enables us to explore the trade-off between the potentially detrimental erosion of local adaptation versus the positive effects of demographic rescue, both of which are facilitated by straying and potentially moderated by the effects of collective navigation.

## 2. Model description and analysis

### (a) Metapopulation framework

We follow the basic framework described by Ronce & Kirkpatrick [12], where dispersal connects two populations *i* and *j* that inhabit two distinct habitats, each with abundances *N*_*i*_ and *N*_*j*_ and trait values *x*_*i*_ and *x*_*j*_, respectively (see table 1 for parameter definitions and values). In our version of the model, *i* and *j* are locally adapted to site-specific conditions such that there is an optimum trait value *θ*_*i*_ and *θ*_*j*_ associated with each habitat, where recruitment is maximized if the trait value of the local population equals the optimum (*x* = *θ*). Moreover, we assumed that *x*_*i*,*j*_ are normally distributed with means *μ*_*i*,*j*_ and have the same standard deviation *σ*. As such, the recruitment rate *R*(*μ*(*t*), *θ*) for both populations is determined by the mean trait value of the local population relative to the optimal value at that site. Mean trait values for both populations are dynamic variables and change over time in response to differences in recruitment as individuals mix between sites. Trait means for each population are thus subject to selection, the strength of which is proportional to the difference between the trait mean and the local trait optimum at a given point in time [12,41,42]. This is broadly consistent with empirical patterns observed in Pacific salmon dynamics [43]. The two populations occur in spatially separate sites that are close enough that a proportion of the population *m* strays into the wrong site. If there is no straying between these populations, then the mean trait evolves towards the optimal value for each site 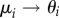, and the recruitment rate for each population is maximized. If there is straying between populations, then the trait means in each respective location will be pulled away from their optima, and recruitment rates will decline. As 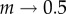, the populations are perfectly mixed.

**Table 1.** Table of parameters, definitions and assigned values or ranges. dyn., dynamic variables.

parameter	definition	value/range
*N*_*i*_(*t*); *N*_*T*_(*t*)	individual; aggregate population over time	dyn.
*x*_*i*_	trait value for an individual in population *i*	dyn.
*μ*_*i*_(*t*)	mean of *x* for population *i* over time	dyn.
*m*; *m*(*t*)	constant; density-dependent straying	(0, 0.5); dyn.
*m*_0_	individual straying probability	(0, 0.5)
*C*	strength of collective behaviour (low = strong)	(10^1^, 10^5^)
*r*_max_	maximum recruitment rate	2.0
*β*	strength of density dependence	10^−3^
*θ*_*i*_	optimal trait value for habitat *i*	5.0
*σ*^2^	genetic variance of trait *x*	1.0
*τ*	strength of selection	1.0
*h*^2^	heritability	0.2
Δ*θ*	habitat heterogeneity	2.0
*ε*	sensitivity of *m*_0_ to changes in Δ*θ*	20.0
PE	portfolio effect	≥1
*T*	terminal simulation time	10^5^

We used the discrete Ricker framework described by Shelton & Mangel [44] as the basis for our two-site metapopulation model, with the added effect that the size of the local population *N*_*i*_ is altered by mixing *mN*_*j*_ individuals from the remote population. Moreover, we assume that there is no demographic overlap between generations, consistent with the life history of many populations of pink salmon (*Oncorhynchus gorbuscha*) that all mature at two years of age and die after one reproductive season. Because total recruitment will be determined by both locals (with a mean trait value closer to the site optimum) and strays (with mean trait values further from the local optimum), the recruitment of the aggregate for population *i* is determined by the mean of the trait mix *R*_*i*_(*ω*_*i*_*μ*_*i*_(*t*) + (1 − *ω*_*i*_)*μ*_*j*_(*t*), *θ*_*i*_), where2.1
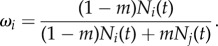
This mix of individuals is subject to identical compensatory effects, which is determined by the parameter *β*. Taken together, the difference equation that determines changes in population size from time *t* to *t* + 1 is2.2
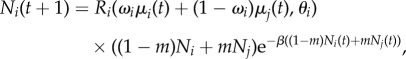
where the recruitment of the population as a function of the mean trait value at time *t* and the local trait optimum is2.3
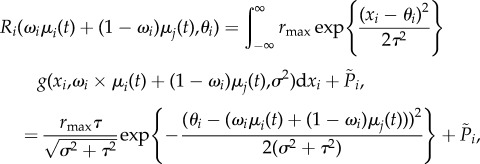
where *g*(*x*_*i*_) is the Gaussian probability density function for the trait *x*_*i*_. The mismatch between the mean of the local trait mix *ω*_*i*_*μ*_*i*_(*t*) + (1 − *ω*_*i*_)*μ*_*j*_(*t*) and the local optimum *θ*_*i*_ scales the recruitment rate for the population, and 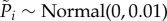 introduces a small amount of demographic stochasticity. The parameter *τ* is the strength of selection and controls the sensitivity of recruitment to changes in the mean trait value away from the optimum. Because straying individuals are emigrating from a population with a mean trait value further from the local optimum, their rate of recruitment is diminished. Recent studies of wild sockeye salmon (*Oncorhynchus nerka*) have indeed found that straying individuals have lower lifetime fitness than individuals that do not stray, although it is unknown at what life stage this selection occurs [38].

Because individuals from the local population are mixed with individuals from the remote population via straying and subsequent reproduction, the resulting trait distribution is a complex mixture of trait distributions. We make two simplifying assumptions. First, we approximate the distribution resulting from the mix of remote and local individuals prior to reproduction as a Gaussian distribution, where *X*_*i*_ = *x*_*i*_ with probability *g*(*x*_*i*_). The expectation of the actual trait distribution as well as the Gaussian approximation are the same, such that E{*X*_*i*_} = *ω*_*i*_*μ*_*i*_ + (1 − *ω*_*i*_)*μ*_*j*_, with weights corresponding to the proportion of the mixed population that are local individuals, *ω*_*i*_, and straying individuals, 1 − *ω*_*i*_. Thus, strays can successfully reproduce and introduce their genotypes into the recipient population, which is supported by observations in wild populations [45]. Second, we assumed that changes in trait variance through time are minimal, such that *σ*^2^ is constant over time, which is a common simplification in eco-evolutionary models of population dynamics [12,42,46–48]. These simplifications are the same as those introduced by Ronce & Kirkpatrick [12], and were shown to have negligible impacts on dynamics.

Following Lande [42], and given our assumption of trait distribution normality, the mean trait value thus changes through time according to the difference equation2.4
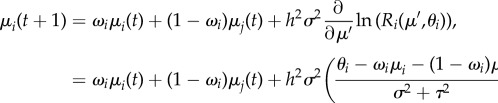
with *μ*′ = *ω*_*i*_*μ*_*i*_(*t*) + (1 − *ω*_*i*_)*μ*_*j*_(*t*). Although trait heritability *h*^2^ among salmonids is variable, most life-history traits have an *h*^2^ < 0.5 [49], and for all additional analyses we have conservatively set *h*^2^ = 0.2. Together, equations (2.2) and (2.4) for two linked populations *i* and *j* define the four-dimensional system of difference equations that describe the eco-evolutionary dynamics of the metapopulation.

### (b) Density-dependent straying

There is mounting evidence that straying is density-dependent, consistent with predictions of the collective navigation hypothesis [23,25]. Specifically, straying has been linked directly to a collective decision-making phenomenon, where greater numbers of individuals tend to decrease the rate at which individuals err, reducing the overall proportion of a population that strays. Following Berdahl *et al.* [21], given the probability that an individual strays is *m*_0_, the proportion of the local population *i* that strays is2.5
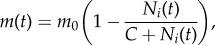
where *C* is a half-saturation constant, determining to what extent collective behaviour, as a function of group size, diminishes straying. For a given *m*_0_, if *C* is small, relatively smaller groups of organisms ‘correct’ for higher individual error rates, suppressing straying between sites. Small values of *C* indicate that the effects of collective behaviour on modifying straying—thus leading to collective navigation—are strong. Henceforth, we refer to *C* as determining the strength of collective behaviour: as 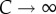, the effect of collective behaviour becomes weaker, such that the size of the population has no impact on straying, and 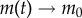. Thus, although the strength of collective behaviour depends both on *C* as well as *m*_0_, for a given *m*_0_, *C* is an effective proxy for the strength of collective behaviour.

### (c) Measuring metapopulation robustness

We evaluated two complementary measures of metapopulation robustness by quantifying (i) the average-CV PE [34,50] and (ii) the recovery time, which is the time required for the system to return to a steady state following an induced disturbance to one or both populations [51]. Throughout, we refer to an increase in PEs and/or reduction in recovery time as promoting metapopulation robustness.

The average-CV PE is, as the name implies, the average CV of the population biomass *N*_*i*_ divided by the CV of the aggregate biomass 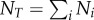 [52], such that2.6
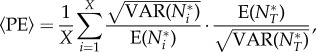
where, in this case, the number of populations is limited to *X* = 2, and the expectations E( · ) and variances V*AR*( · ) are evaluated at the steady state, denoted by ‘*’. As the CV of N*_T_ decreases relative to that of the constituent populations, 〈PE〉 > 1, and the metapopulation is presumed to be more stable because the aggregate has functioned to dampen population-level variance. Moreover, PEs greater than unity correspond to less synchronization [34,53,54] and thus a greater potential for demographic rescue among populations, buffering the system as a whole against extinction.

A more direct way to measure system robustness is to measure the time required for the system (measured as the aggregate steady-state biomass N*_T_) to recover following an induced disturbance: systems that recover quickly (shorter recovery times) are more robust than those that recover more slowly (longer recovery times). Although there is a direct relationship between the rate of return following a small pulse perturbation and the magnitude of the leading eigenvalue of the Jacobian matrix [55], because we aimed to (1) assess the effects of a large perturbation far from the steady state, and (2) estimate the time required for all transient effects to decay following this perturbation, we used a simulation-based numerical procedure. Recovery time was calculated by initiating a disturbance at *t* = *t*_d_, and monitoring *N*_*T*_(*t*_d_ + *t*) as 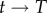, where *T* is large. The aggregate was deemed recovered at *t*_r_, such that the recovery time was calculated as *t*_r_ − *t*_d_, and recovery at *t* = *t*_r_ was determined by the initial *t* where *N*_*T*_(*t*) < E(N*_T_) ± SD(N*_T_) for *t*∈(*t*_r_, *T*), where SD( · ) is the standard deviation (illustrated in electronic supplementary material, figure S1). If the system recovers to a different basin of attraction after the perturbation is applied, the recovery time is calculated with respect to the newly acquired steady state.

Numerically estimating the time that it takes for a perturbed system to recover also permits a more nuanced perspective of metapopulation robustness. For example, if populations settle to alternative stable states, comparing recovery times after a disturbance applied to individual populations allows for an assessment of which component of the metapopulation has a longer-lasting influence on the system's recovery. We measured recovery time following three types of induced disturbance: (i) extinction of the low-density population; (ii) extinction of the high-density population (scenarios (i) and (ii) are equivalent if populations have the same density); (iii) near-collapse of both populations where just 1.0% of each survives.

## 3. Results

**Figure 1.**
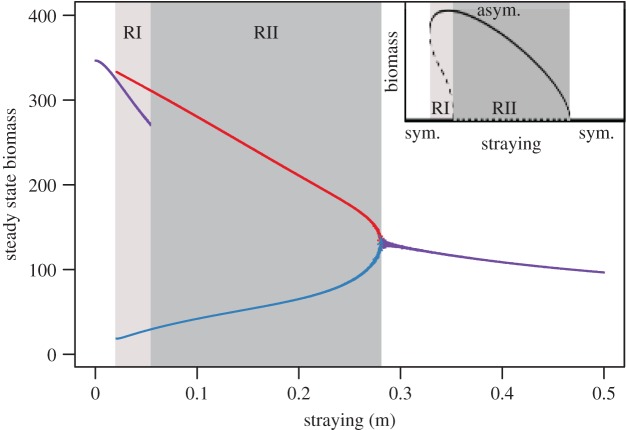
The steady-state densities of *N*_*i*_ and *N*_*j*_ versus straying *m* for the constant straying model. Alternative stable states exist for regimes I and II, labelled RI and RII, respectively. In regime I, the system can approach qualitatively different states: a symmetric, intermediate state (purple), and asymmetric dominant (red) and subordinate (blue) states. In regime II, only one type of attractor exists: an asymmetric dominant/subordinate state (red and blue points, respectively), and its mirror image where identities of dominant and subordinate are exchanged. Inset: a qualitative sketch of the bifurcation diagram, showing the stable (solid lines) and unstable (dashed lines) fixed points in regimes I (light grey area) and II (dark grey area). The symmetric condition (sym.) is the horizontal line at the base of the inset, whereas the asymmetric condition (asym.) is represented by the curved line. (Online version in colour.)

At low values of density-independent straying the system approaches a fixed point at which both populations persist at equal population size, but as we increase straying, other fixed points are created in which the population sizes are asymmetric (figure 1, inset). The system's underlying symmetry implies that for every asymmetric fixed point there must be another ‘mirror-image’ fixed point in which we find the same population sizes, but where the identities of the populations are reversed. Asymmetric fixed points appear in bifurcations as a critical value of the straying parameter is crossed. As the noise in the system is negligible, for the purposes of the bifurcation diagram we can use concepts of deterministic bifurcation theory. Based on the Jacobian eigenvalues, we conjecture that these bifurcations are fold bifurcations. In a generic fold bifurcation of maps, two new fixed points are created, one of which is unstable, while the other is stable [55]. In this case, two of these bifurcations occur at the same time, one that creates fixed points where the first population is dominant in the stable fixed point, whereas the second bifurcation creates the mirror-image fixed points where the second population is dominant.

In the asymmetric states, the dominant population is well-adapted and has a high rate of recruitment. The (small) fraction of this population that strays to the subordinate site constitutes a considerable inflow of individuals, such that the population in the subordinate site is not as well-adapted. This in turn reduces reproduction and stabilizes the asymmetry. In a regime found where straying is low (regime I), both the symmetric and the asymmetric states are stable fixed points of the system (figure 1). Which of these fixed points is approached depends on the initial conditions.

As we increase straying, the asymmetric states eventually collide with the stable symmetric state. A subcritical pitchfork bifurcation occurs in which the unstable asymmetric states vanish and the symmetric state is destabilized. After this bifurcation, the stable asymmetric states are the only attractors. We find a wide regime (regime II) where the system will always approach an asymmetric state where one population is suppressed. However, if straying is increased further, the imbalance in population sizes becomes harder to maintain. Eventually, we reach a critical point where the stable asymmetric fixed points become symmetric and collide with the unstable symmetric fixed point. The system undergoes a supercritical pitchfork bifurcation, in which the stable asymmetric fixed points vanish, while the unstable symmetric fixed point is stabilized. After this bifurcation, the symmetric fixed point is the only attractor in the system. Importantly, we find that increasing the asymmetry in the vital rates of populations between sites does not significantly alter the presence or position of these different regimes (see electronic supplementary material, figure S2).

### (a) Nonlinear effects of straying on metapopulation robustness

**Figure 2.**
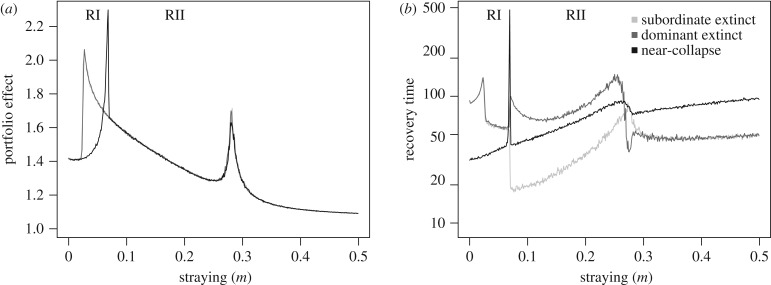
Measures of metapopulation robustness for the constant straying model as a function of straying *m*. Alternative stable state regimes I and II corresponding to those in figure 1 are labelled RI and RII, respectively. (*a*) Portfolio effect as a function of *m*. (*b*) Recovery time as a function of *m*. Measures of metapopulation robustness are shown with respect to different induced disturbances: the near-collapse of both populations (black), and the lone extinction of either the dominant (dark grey) or subordinate (light grey) population. Portfolio effects are different for the near-collapse and single extinction scenarios due to different CVs for the populations and aggregate in alternative basins of attraction that exist in regimes I and II.

Straying has a large effect on metapopulation robustness, measured by the PE and the time to recovery following the three types of induced disturbance: near-collapse of both populations, the extinction of the dominant population and the extinction of the subordinate population (figure 2). Importantly, the presence of alternative stable state regimes I and II both have a direct impact on robustness as a function of straying *m*. We observe that as straying increases, the PE increases sharply as regime I or II is entered, and then declines gradually (figure 2*a*). Thus, low levels of straying (2–10% of the population) are associated with the strongest PEs.

Different types of disturbance lead to different relationships between straying and PEs. When either population suffers extinction, the PE is shown to increase with lower straying; in the case of near-collapse of both populations, PE increases when straying is higher (figure 2*a*). This difference is due to the hidden basin of attraction at low population densities that only plays a role when a disturbance impacts a single population. In other words, disturbance to a single population can push that population into a low-density alternative state, which in turn contributes to higher PE. The increase in PE for the synchronous near-collapse scenario occurs at higher values of *m* when the system enters regime II, where there exists only an asymmetric dominant (high-density) and subordinate (low-density) state. The PE spikes again when straying is very high and the system leaves regime II, entering a symmetric low-density state.

Similar patterns are observed with respect to the recovery time as straying is increased (figure 2*b*). For lower *m*, recovery following individual extinctions is impacted by the appearance of low-density basins of attraction in regime I, whereas recovery following near-collapse is not. For intermediate values of *m* (regime II), the time to recovery is only diminished when the subordinate population becomes extinct, whereas the time to recovery following near-collapse and the extinction of the dominant population are similar and grow until regime II is exited at high *m*. In the case where the subordinate population is extirpated, the most rapid recovery occurs when straying is low (*m* = 0.08). By contrast, when the dominant population goes extinct, the most rapid recovery is associated with minimal straying. It should be noted that when there is no straying (*m* = 0), recovery time is infinite and these values are not shown. Increased straying generally leads to longer recovery times when both populations suffer near-collapse.

Collectively, these patterns in recovery time and PE are influenced by the different alternative stable state regimes. As the alternative stable state regime is approached with increasing *m*, both measures of robustness increase sharply due to an amplification in variance within both populations. This amplification in variance is the product of *critical slowing down*, which occurs near some bifurcations [56] and has been suggested to serve as an early warning indicator for approaching phase transitions [56–60]. At this point, PE peaks along with recovery time, suggesting the former is not a good indicator of robustness very close to the bifurcation. Because these large increases in PE and recovery time pertain to a very small range of *m*, we do not consider them to be biologically relevant, and they are primarily useful in this context for observing transitions between dynamic regimes. In general, high PE corresponds to shorter recovery times, and low PE corresponds to longer recovery times (see electronic supplementary material, figure S3). Together, these results suggest that under the assumption of constant (and symmetric) dispersal, robustness depends strongly on the magnitude of straying as well as the type of disturbance experienced by the metapopulation. We next examine how density-dependent straying challenges these expectations.

### (b) The effects of collective navigation and density-dependent straying

**Figure 3.**
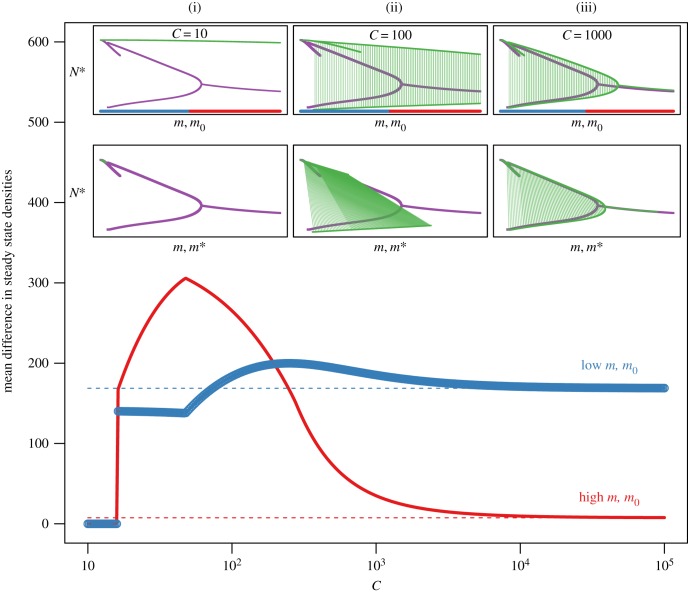
Comparison of steady-state population densities for the constant straying model and density-dependent straying model. Inset: steady-state densities for the constant straying model (purple) and density-dependent straying model (green) for different strengths of collective behaviour. Low *C* corresponds to strong effects of collective behaviour. The top row shows steady-state densities as a function of individual straying *m*_0_; the bottom row shows steady-state densities as a function of straying at the steady state m*. Vertical green lines link paired subordinate and dominant population densities. Main: The absolute difference in steady-state densities averaged across intervals of low straying (0 < *m*, *m*_0_ < 0.25; blue) and high straying (0.25 < *m*, *m*_0_ < 0.5; red). Horizontal dashed lines correspond to the mean absolute differences in steady-state densities for low (blue) and high (red) density-independent straying. As 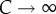, mean absolute differences in steady-state densities become equivalent.

When collective behaviour is very strong (small values of *C*), small increases in population density beget large reductions in straying. These reductions can be large enough that the system avoids the alternative stable state regime altogether (figure 3, left inset (i)). Conversely, when collective behaviour is very weak, such that *C* is very high, there is effectively no reduction in straying with increased group size, and the dynamics are those expected if straying were constant (figure 3, right inset (iii)). However, when collective behaviour is of intermediate strength (10^2^ ≲ *C* ≲ 10^3^), the dynamics are altered in two important ways. First, in the alternative stable state regime, the low-density subordinate population has correspondingly higher m* (where m* is density-dependent straying at the steady state), whereas the high-density dominant population has correspondingly lower m* (figure 3, centre inset (ii)). Second, the alternative stable state regime results in a ΔN* that is reduced or negligible when individual straying is low, and magnified when individual straying is high (figure 3, main). In other words, when collective behaviour is of intermediate strength—the more realistic range for species that navigate via collective decision-making—increased individual straying exaggerates the differences between the steady-state densities, effectively pushing the subordinate population closer to extinction.

**Figure 4.**
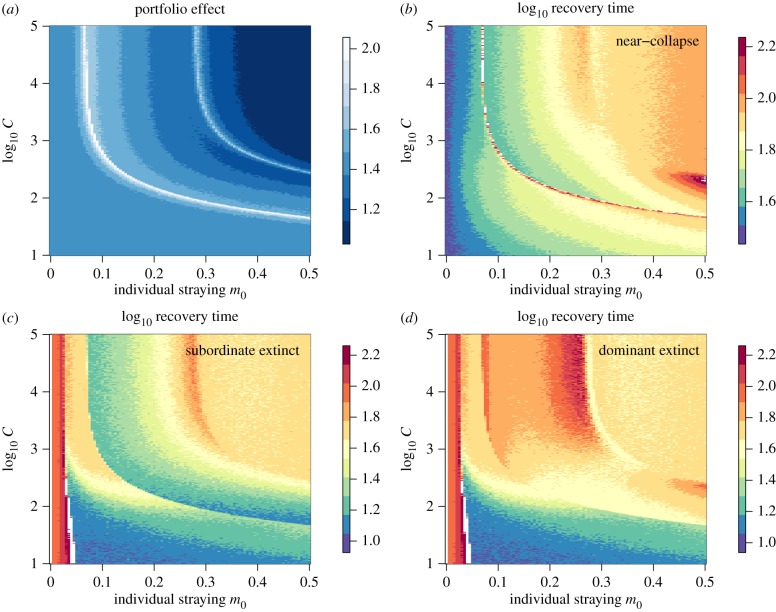
Measures of metapopulation robustness for the density-dependent straying model as a function of individual straying *m*_0_ and the strength of collective behaviour *C* (note the l*og*_10_ scale, including (*a*) the portfolio effect, (*b*) the time to recovery following near-collapse of both populations, (*c*) the time to recovery following the extinction of the subordinate population and (*d*) the time to recovery following the extinction of the dominant population. (Online version in colour.)

Density-dependent straying directly alters the dynamic regimes of the model, and this has a large effect on metapopulation robustness. When the effects of collective behaviour are weak (high *C*), the PEs and recovery times conform to those examined in the case of constant straying (figure 4; cf. figure 2). When the effects of collective behaviour are very strong (low *C*), we observe that recovery times are shorter in the case of near-collapse (figure 4*b*). Recovery times also tend to be shorter when a single population goes extinct except for at very low *m*_0_, in which case the time to recovery is much longer (figure 4*c*,*d*). As before, when the strength of collective behaviour is intermediate (10^2^ ≲ *C* ≲ 10^3^), the relationships are more complex. With intermediate *C* there is a general increase in robustness if straying is low–intermediate (higher PE, shorter recovery time), followed by an erosion in robustness as straying becomes high. In this parameter space, collective navigation results in the low-density population that is straying more, losing well-adapted local individuals, while still receiving some maladapted strays from the larger population, thereby increasing the likelihood of stochastic extinction.

**Figure 5.**
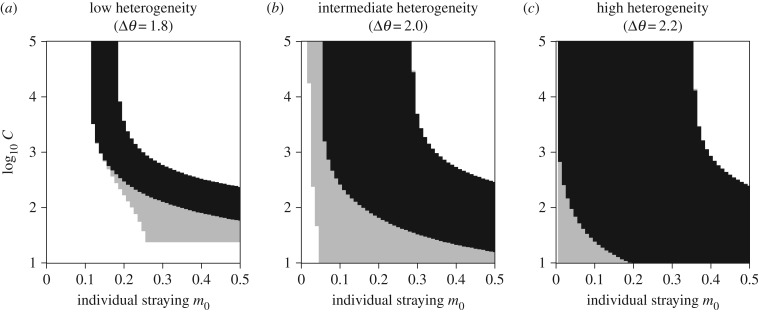
Alternative stable state regimes I (grey) and II (black) as a function of individual straying *m*_0_ and the strength of collective behaviour *C* (note the l*og*_10_ scale). Regime I signifies parameter space where there is either (1) an intermediate-density, symmetric steady state, or (2) an asymmetric dominant/subordinate density. Regime II signifies parameter space where there is an asymmetric dominant/subordinate steady-state density. The white space to the left (lower values of *m*_0_) signifies high-density, symmetric steady states, and the white space to the right (higher values of *m*_0_) signifies low-density, symmetric, steady states. Relationships are shown for (*a*) low habitat heterogeneity (Δ*θ*), (*b*) intermediate habitat heterogeneity and (*c*) high heterogeneity. The horizontal cut-off of Region I at low values of *C* in (*a*) is due to numerical limitations.

Sharp changes in metapopulation robustness are due to changes in alternative stable state regimes I and II as the strength of collective behaviour increases (lower *C*, figure 5). When collective behaviour is weak (large *C*), alternative stable states tend to occur at low–intermediate values of individual straying *m*_0_. As collective behaviour is strengthened (smaller *C*), regime II is avoided at lower values of *m*_0_ and expands at higher values of *m*_0_. When the effects of collective behaviour are very strong, regime II collapses (black region in figure 5*b*) and gives way to regime I, which plays a larger role over a larger range of *m*_0_ when *C* is low (grey region in figure 5*b*). Importantly, when the strength of collective behaviour is intermediate, both regimes I and II are relevant at low–intermediate *m*_0_.

### (c) The role of habitat heterogeneity and changing selective landscapes

As habitat heterogeneity (Δ*θ*) increases, even small amounts of straying can lead to the appearance of alternative stable states. However, if straying is density-dependent, the strength of collective behaviour has a large influence on the occurrence of both alternative stable state regimes I and II. When heterogeneity is low and the effects of collective behaviour are weak such that straying is constant (high *C*), regime II occurs for small–intermediate *m*_0_, and regime I does not play a role (figure 5*a*). The absence of regime I implies that there are no hidden steady-state configurations that might trap a disturbed population in an asymmetric low-density state. As the strength of collective behaviour increases, regime I appears at a cusp and becomes increasingly dominant with greater individual straying. For sites distributed across more heterogeneous habitats, the alternative stable state regimes I and II expand (figure 5*b*,*c*). Regime II dominates at all but very high individual straying when the effects of collective behaviour are weak (high *C*) and very low individual straying when the effects of collective behaviour are strong (low *C*). Moreover, in highly heterogeneous habitats, if the effects of collective behaviour are strong and straying is low (low *m*_0_ and low *C*), regime I, which harbours low-density basins of attraction, cannot be avoided.

**Figure 6.**
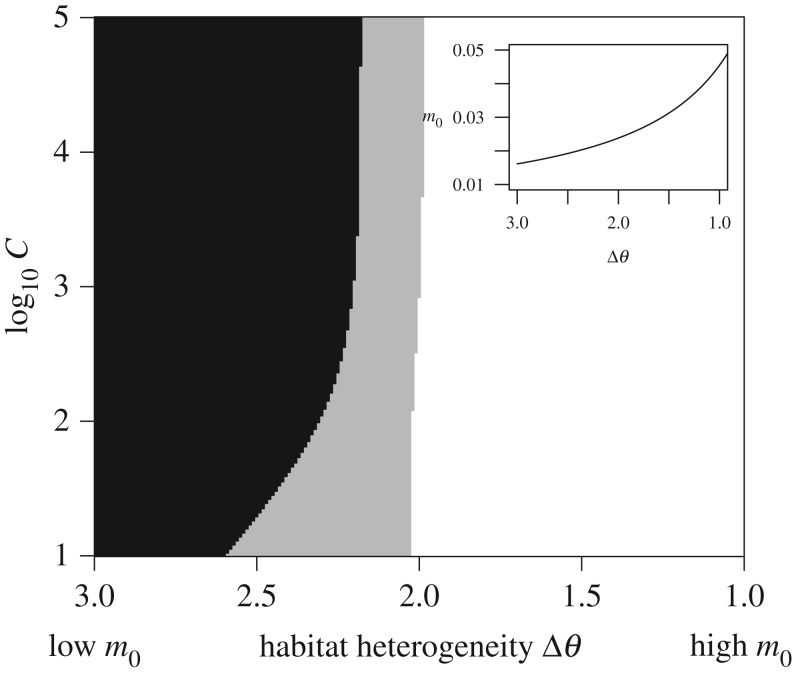
Alternative stable state regimes I (grey) and II (black) as a function of individual straying *m*_0_ and the strength of collective behaviour *C* (note the l*og*_10_ scale), for the case where individual straying increases with lower habitat heterogeneity (inset). Regime I signifies parameter space where there is either (1) an intermediate-density, symmetric steady state, or (2) an asymmetric dominant/subordinate density. Regime II signifies parameter space where there is an asymmetric dominant/subordinate steady-state density.

Until now, we have treated straying and habitat heterogeneity as independent parameters; however, they could covary. For instance, if sites are separated by greater distance, they may be assumed to have increased habitat heterogeneity as well as less straying. Alternatively, individuals may be genetically predisposed to stray into sites that are more similar [38,61], such that higher straying can be assumed to occur between sites that are more homogeneous in aspect. We implemented this inverse relationship by setting *m*_0_ = 1/(2 + *ε*Δ*θ*) where *ε* controls the degree to which an increase in Δ*θ* lowers *m*_0_ (figure 6, inset). Accordingly, *m*_0_ is increased for lower Δ*θ* and decreased for higher Δ*θ* , such that there is less straying between dissimilar sites and more straying between similar sites. Under these conditions, we find that regime II appears for very low *m*_0_, and regime I appears for higher *m*_0_ (figure 6), which is opposite the case where *m*_0_ and Δ*θ* are independent. In this case, as straying increases and Δ*θ* decreases, a single (symmetric) steady-state emerges as the fold bifurcation is crossed.

## 4. Discussion

In this paper, we show that density-dependent straying between populations consistent with collective navigation, coupled with localized selection against immigrant phenotypes, has large, nonlinear impacts on metapopulation robustness. Building upon the dynamical framework introduced by Ronce & Kirkpatrick [12], we assess robustness by measuring (1) the average-CV PE [4,52], a statistical metric commonly used to assess the buffering capacity of metapopulations, and (2) the recovery time, defined here as the time required for the aggregate metapopulation biomass *N*_*T*_ to return to its steady state following an induced disturbance, which is mechanistically linked to persistence [51]. These statistical and mechanistic descriptors of metapopulation dynamics and robustness are tightly coupled (electronic supplementary material, figure S3), which is not uncommon for diverse metrics of stability [62]. We introduce density-dependent straying by assuming that larger group sizes lower population-level straying from the baseline probability than an individual errs *m*_0_, with the strength of this effect determined by *C* in equation (2.5) (lower values of *C* indicate that the effects of collective behaviour are strong). Generally, we find that when the effects of collective behaviour are strong such that collective navigation occurs, metapopulation robustness is enhanced. However, empirical observations of natural populations suggest that the effects of collective behaviour are intermediate (e.g. 10^2^ ≲ *C* ≲ 10^3^) [21,25]. In this case, we find that the robustness of the metapopulation is increased only if the probability that individuals stray is low, and is substantially eroded if the probability that individuals err is high.

Metapopulation robustness was found to depend strongly on the magnitude of straying between sites. We generally found that metapopulation robustness was highest (as indicated by higher PE and lower recovery times) when straying was at a low–intermediate level. A central dynamic of the model is that straying can lead to the emergence of asymmetric alternative stable states, or *migrational meltdown* [12], pushing one of the populations to a dominant, well-adapted, high-density state, and one to a subordinate, maladapted, low-density state. Although there are subtle differences in our model from the framework presented by Ronce & Kirkpatrick [12], the general dynamic features are the same if we assume that dispersal is symmetric between sites and density-independent (which occurs when 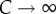). The dynamic regimes that emerge from the eco-evolutionary model—in particular, the occurrence of alternative stable state regimes I and II (figure 1)—have large effects on both the PE and the recovery time following an induced disturbance (figure 4). In general, we find that intermediate straying increases the PE and lowers the time to recovery, particularly in the case of the extinction of the subordinate (low-density) population. In this case, elevated PE occurs when the system enters either regime I or II (depending on the initial conditions), where one population assumes a subordinate low-density state. Given that the time to recovery following near-collapse of both populations increases with straying (figure 4*b*), it would suggest that all but the lowest values of density-independent straying erode robustness, regardless of the increase in PE observed at more intermediate values.

This themed issue formalizes the role of collective movement in the ecology of natural systems and illuminates a signature of collective navigation in animal populations on the move. Here, we explore the implications of this collective navigation for metapopulations. We highlight three important findings that contribute to our understanding of collective movement, suggesting that density-dependent straying may play an important role in the persistence of metapopulations over evolutionary time.

First, if the effects of collective behaviour are very strong (low *C*), metapopulation robustness is increased, due primarily to the avoidance of alternative stable state regime II (figures 4 and 5*b*). This means that—despite potentially high individual error rates—group formation minimizes straying. This occurs when groups of less than or equal to 100 individuals significantly minimize straying, which is probably unrealistic. Moreover, when the effects of collective behaviour are strong, regime II gives way to the dominance of regime I, which harbours low-density basins of attraction (figure 1). The presence of low-density basins of attraction can effectively trap disturbed populations in a subordinate steady state, not unlike the Allee effects observed in the collective migration model explored by Berdahl *et al*. [21].

Our second important finding reveals that when the effects of collective behaviour are intermediate, metapopulation robustness is impacted in three ways, depending on the magnitude of individual straying. Here, the system is generally in alternative stable-state regime I or II except for perhaps unrealistically low levels of individual straying (figure 5*b*). If individual straying is high (*m*_0_ > 0.25), (1) there is a magnified difference between the numerical densities of the subordinate and dominant populations, effectively pushing the subordinate population to lower steady-state densities (figure 3); (2) the PE is low, such that the CV for the aggregate metapopulation biomass is on par with the CV for its constituent populations; and (3) more time is generally required for the population(s) to recover following an induced disturbance, and this is particularly true for the recovery of the system following near-collapse of both populations (figure 4*b*). Together, this suggests that when the effects of collective behaviour are intermediate, and straying is high, there is an overall reduction in metapopulation robustness, thereby reducing persistence.

Empirical observations of straying support low–intermediate levels of individual error rates in most species [16,17]. If *m*_0_ is low (*m*_0_ < 0.25), (1) alternative stable state regime II tends to be avoided for a larger range of *m*_0_ (figures 3 and 5*b*); (2) the PE is exaggerated, meaning that the metapopulation has dampened variance relative to its constituent populations (figure 4*a*); and (3) the time required for the population(s) to recover following an induced disturbance is lower (figure 4*b*). Interestingly, the largest PEs are observed when straying is just large enough to enter regime II, where one population assumes a subordinate state, and the differences between the subordinate and dominant population densities are largest. This does not appear, in fact, to be a robust condition because the system relies to a large extent on the dominant population as the source, whereas the subordinate population assumes the role of a sink. However, recovery time was measured with respect to the aggregate biomass of the metapopulation (*N*_*T*_ = *N*_*i*_ + *N*_*j*_), and despite the source–sink dynamics that emerge in regime II, the aggregate biomass of the system recovers more quickly in this region following the near-collapse of both populations (figure 4*b*). From this perspective, the existence of asymmetric dominant/subordinate alternative stable states could be considered to be more robust with respect to the recovery time of the total biomass, or less robust because one population is always at greater risk of stochastic extinction.

Third, we find that greater habitat heterogeneity increases the role of alternative stable state regimes, particularly when the effects of collective behaviour are strong (high Δ*θ*, low *C*; figure 5*c*), and this increases the potential complexity of metapopulation dynamics. Salmon are distributed and stray across a diverse range of habitats, and the rates of straying between geographically diverse sites can be plastic and idiosyncratic [36]. Our surrogate measure for habitat heterogeneity is the difference in trait optima between sites Δ*θ*. We show that as habitat heterogeneity increases, the occurrence of alternative stable states associated with regime II becomes unavoidable, particularly for 0.1 ≤ *m*_0_ ≤ 0.4, and regime I is minimized. This may be particularly consequential for populations that are spatially adjacent but separated by sharp environmental boundaries, such that trait optima are divergent yet dispersal is relatively high. Such a scenario plays out repeatedly in the context of interactions between wild and hatchery-produced salmon. Although wild and hatchery populations may occur close on the landscape, and indeed are often sympatric within the same river network, the selective environments to which they are locally adapted differ dramatically [63]. Straying of domesticated hatchery-produced fish from release sites and spawning in the wild reduce the productivity of wild populations through competition and outbreeding depression [64,65].

In other cases, habitats that are closer in space can be assumed to have greater similarity in environmental conditions than those that are geographically distant, and phenotypes of more proximately located populations should be more similar [43,66,67]. It is thus reasonable to expect a larger number of straying individuals between sites that are geographically proximate and indeed evidence corroborates this prediction [68,69]. Alternatively, salmon that cue to specific environmental conditions may be more likely to stray into sites that are structurally and physiognamically more similar [38]. These considerations justify imposing a negative correlation between habitat heterogeneity and individual straying: as site heterogeneity increases, so too should individual straying decrease (figure 6, inset). When habitat heterogeneity and individual straying are linked in this way, we show that very small amounts of individual straying give rise to regime II, and that regime I occurs for higher values of *m*_0_ (figure 6). This pattern is opposite that observed for scenarios where habitat heterogeneity and straying are assumed to be independent, and suggests that increases in straying that are associated with growing similarities between habitats can push a metapopulation into a regime where hidden low-density basins of attraction exist. Thus, management activities that alter dispersal rates by outplanting individuals or reconnecting disconnected habitats could have complex eco-evolutionary consequences [70,71], and compromise management or conservation objectives.

A general message from our theoretical framework is that the emergence of alternative eco-evolutionary states depends jointly on the strength of collective behaviour and level of individual straying, and that this has large implications for metapopulation robustness. Although robustness is in many cases aided by increasing the strength of collective behaviour, the greater role of both alternative stable state regimes I and II portends additional complexity in eco-evolutionary dynamics, and this could serve to hinder effective management. Moreover, this increased complexity at empirically observed levels of straying [16] and at realistic (intermediate) ranges for the strength of collective behaviour is only magnified with increasing habitat heterogeneity and when heterogeneity itself is linked to individual straying. Additional issues that we have not explored here, but that may be particularly relevant to consider, are the effects of including additional sites within the metapopulation network, as well as alternative patterns of dispersal that connect these sites. The structure of dispersal has been shown to have a large influence on population dynamics [54,72–74], and to what extent density-dependent straying influences the eco-evolutionary dynamics of populations in large spatially structured networks is of considerable interest. We are hopeful that these predictions will inspire future theoretical and empirical studies that aim to expand upon the relationships that we have explored.

A particularly salient finding of our work was that density-dependent straying may serve to promote or inhibit population robustness, depending on the strength of the collective behaviour and the underlying magnitude of straying. Salmon have evolved within the context of dynamic geomorphic landscapes where habitat quantity and quality shift as a mosaic through time [75]. Our results provide evidence supporting the hypothesis described in Berdahl *et al.* [25] that collective behaviour may support rapid habitat colonization following natural disturbance such as volcanic eruptions [76], reconnected habitats following restoration [71,77], or in the context of glacial retreat and climate warming [78]. Moreover, our results are consistent with the role of collective behaviour in facilitating reproductive isolation and local adaptation to site-specific selection in populations that recover following disturbance to the extent that straying decreases as population sizes increase. Additionally, collective behaviour may be beneficial in facilitating navigation though increasingly modified and fragmented habitats [23]. On the other hand, collective behaviour coupled with high straying may push populations to extirpation. Thus, collective behaviour could provide both resilience to salmon metapopulations but also vulnerabilities.

Our study broadly indicates that management activities that alter patterns of straying could have profound implications for metapopulation robustness and adaptive potential. High rates of straying are predicted to decrease metapopulation robustness, and there are a series of common practices in salmon management that may be elevating straying rates [26,79]. For example, transporting young salmon downstream to increase survival during outmigration may disrupt the processes involved with critical periods of imprinting prior to or during downstream migration by sea-going individuals and increase straying by adults later in life. Our results support the conservation concern that large-scale releases of salmon produced in hatcheries that stray could decrease robustness of salmon metapopulations through the erosion of PEs and increase in recovery times. Moreover, hatchery environments are associated with marked changes in fish social behaviour that may increase collective dynamics of migrating groups [80], consistent with the findings of Jonsson & Jonsson [30] who report stronger associations between straying and abundance in escaped aquaculture-produced Atlantic salmon than their wild counterparts. Thus, management activities that have the unintended consequence of altering straying may compromise recovery efforts.

Beyond salmon, density-dependent dispersal, whether it is caused by collective decision-making or other factors, has a large influence on the dynamics of populations in the presence of local adaptation. The rate at which individuals err, and the influence of group size on navigation at the population level, are two important components of dynamic dispersal [21]. We show that changes in these characteristics can alter the occurrence and positioning of two different alternative stable state regimes, one of which may harbour hidden low-density basins of attractions that can effectively trap populations after large disturbances. Generally, increasing the strength of collective behaviour mitigates the potentially negative impacts of so-called *migrational meltdown* [12]. Thus, preserving the biological processes that facilitate collective behaviour of migratory species may be an important conservation target in its own right, echoing the sentiments of Hardesty-Moore *et al.* [22]. We suggest that an increased understanding of the proximate and ultimate factors governing dispersal among local populations within metapopulations, across heterogeneous environments, in tandem with the mosaic of selective forces acting on those environments, may be key to promoting persistence in the wild [81].

## Supplementary Material

Supplementary Materials: Eco-evolutionary dynamics, density-dependent dispersal, and collective behavior: implications for salmon metapopulation robustness

## Data accessibility

Code is made available at https://github.com/jdyeakel/SalmonStrays.
