# Supplementary material for: Eco-evolutionary dynamics, density-dependent dispersal and collective behaviour: implications for salmon metapopulation robustness
Source: Philos Trans R Soc Lond B Biol Sci. 2018 Mar 26;373(1746):20170018. doi: 10.1098/rstb.2017.0018 (PMC5882987; doi:10.1098/rstb.2017.0018)
Supplement: Supplementary Materials: Eco-evolutionary dynamics, density-dependent dispersal, and collective behavior: implications for salmon metapopulation robustness [file rstb20170018supp1.pdf]

Supplementary Materials: Eco-evolutionary dynamics, density-dependent dispersal and collective behaviour: implications for salmon metapopulation robustness

We aim to understand how straying between populations may affect phenotypic evolution, and how this evolutionary change may in turn lead to changes in ecological dynamics as they unfold. To do so, we build upon Lande's classic formulation of phenotypic evolution [2]. In what follows, we 1) re-derive Lande's phenotypic evolution equation for one population to introduce the reader to the main concepts and assumptions of the framework used, and, 2) explain how we used this framework for the case of two populations connected by dispersal.

I. Phenotypic evolution from one to two sites

Trait evolution in one site To keep track of the evolution of a phenotypic trait x with probability density function p and mean $\overline{x}$ and variance σ^2, we need a few basic ingredients. First, we define the mean fitness,$\overline{W}$ of the population of individuals bearing the trait x as:

$$\overline{W} = \int p(x)W(x)\,dx. \tag{1}$$

We also define the mean phenotype in the population as

$$\overline{x} = \int xp(x)\,dx, \tag{2}$$

and the mean phenotype after selection (but before reproduction), $\overline{x}_w$ [1], as:

$$\overline{x}_w = \frac{1}{\overline{W}}\int xp(x)W(x)\,dx. \tag{3}$$

Then, we can assess how the change in the phenotype x occurs over time using

$$\Delta x = x(t+1) - x(t), \tag{4}$$

which can be rewritten as:

$$\Delta x = h^2(\overline{x}_w - x(t)), \tag{5}$$

where h^2 is the heritability of the trait, and the difference $\overline{x}_w - x(t)$ is the classical "selection differential" [1, 2]. In other words, 5 is none other that the Breeder's equation.

To understand how x changes over time, we first need to understand how the mean fitness of the population changes with a change in the mean trait value or $\frac{\partial \overline{W}}{\partial \overline{x}}$ (i.e. the shape of the "adaptive landscape"). Using 1 we have:

$$\frac{\partial \overline{W}}{\partial \overline{x}} = \frac{\partial}{\partial \overline{x}}\int p(x)W(x)\,dx. \tag{6}$$

Using Leibniz rule we can pass the derivative under the integral sign,

$$\frac{\partial \overline{W}}{\partial \overline{x}} = \int \frac{\partial p(x)}{\partial \overline{x}} W(x)\,dx, \tag{7}$$

and assuming p is Gaussian (i.e. of the form $\frac{1}{\sqrt{2\pi\sigma^2}}e^{-\frac{1}{2}\frac{(x-\overline{x})^2}{\sigma^2}}$), we calculate the derivative of p with respect to $\overline{x}$, which leads to:

$$\frac{\partial \overline{W}}{\partial \overline{x}} = \int \Big[\frac{x-\overline{x}}{\sigma^2}\Big] p(x)W(x)\,dx. \tag{8}$$

We can now expand in 8 to get,

$$\frac{\partial \overline{W}}{\partial \overline{x}} = \frac{1}{\sigma^2}\int xp(x)W(x)\,dx - \frac{\overline{x}}{\sigma^2}\int p(x)W(x)\,dx, \tag{9}$$

which making use of equations 1 and 3, becomes $\frac{\partial \overline{W}}{\partial \overline{x}} = \frac{\overline{W}}{\sigma^2}\overline{x}_w - \frac{\overline{x}}{\sigma^2}\overline{W}$, and can be factored as $\frac{\partial \overline{W}}{\partial \overline{x}} = \frac{\overline{W}}{\sigma^2}(\overline{x}_w - \overline{x})$. Using 5, we can rearrange this expression to obtain an equation that relates the change in the average phenotype from one time step to the next to the amount of heritable variation, $\sigma^2 h^2$, and the adaptive landscape $\frac{\partial \overline{W}}{\partial \overline{x}}$:

$$\Delta \overline{x} = \frac{\sigma^2 h^2}{\overline{W}}\frac{\partial \overline{W}}{\partial \overline{x}} = \sigma^2 h^2 \frac{\partial \ln \overline{W}}{\partial \overline{x}}. \tag{10}$$

Equation 10 (Lande 1976's equation 7), is a staple of evolutionary biology. It is also possible to use 10 and 4 to write a recurrence relationship that allows to simulating evolutionary change over time in a trait x as:

$$\overline{x}(t+1) = \overline{x}(t) + \sigma^2 h^2 \frac{\partial \ln \overline{W}}{\partial \overline{x}}. \tag{11}$$

Equation 11 thus assumes the existence of one population with a normally distributed trait in which selection occurs before reproduction, the probability of surviving to reproduce depends on the value of the trait different individuals have, and that the trait is normally distributed after reproduction.

Expanding the single site approach to two sites linked by dispersal In what follows we show how Lande's approach can be used to calculate the magnitude of the phenotypic change between two populations with one trait with two different means in each site. We will then 1) show why it is not necessarily useful to use the obtained expression in a simulation context, and 2) introduce an approximation that retains the features important for exploring how trait and population dynamics impact each other in the case of dispersing populations.

We explore the case of two populations that exchange migrants, with a normally distributed trait x that controls recruitment (as explained in the main text), with each population having a different mean, μ_i and μ_j, and same variance σ^2. For simplicity, we will focus on a local population i relative to the connected population j.

When individuals from population j stray into population i, and individuals of population i stray into population j, the phenotypic distribution of trait x in population i is a mixture distribution, $p(x)$, of the form:

$$p(x) = \omega_i g(x, \mu_i) + (1-\omega_i) g(x, \mu_j). \tag{12}$$

where $g(\cdot)$ is the Gaussian probability density function, and ω_i is the proportion of the mixed population that is composed of individuals local to site i relative to those that have dispersed into site i from site j. This distribution has a mean $\overline{x} = \omega_i\mu_i + (1-\omega_i)\mu_j$. As before, we define the mean fitness as in equation 1, only that now p is a mixture distribution. Let us use Lande's approach to obtain an expression for the change in the mean phenotype of the mixture distribution from one time step to the next. We thus ask, what is the change in $\overline{W}$ with a change in $\overline{x}$? We can use Lande's approach [2] to derive equation 7, replacing $p(x)$ by the mixture (equation 12) to obtain

$$\frac{\partial \overline{W}}{\partial \overline{x}} = \int \frac{\partial(\omega_i g(x,\mu_i) + (1-\omega_i) g(x,\mu_j))}{\partial \overline{x}} W(x)\,dx. \tag{13}$$

To obtain the derivative of equation 13, we introduce a change in variables, which is provided by the fact that $\overline{x} = \omega_i\mu_i + (1-\omega_i)\mu_j$:

$$\begin{cases} \mu_i = \frac{\overline{x}-(1-\omega_i)\mu_j}{\omega_i} \\ \mu_j = \frac{\overline{x}-\omega_i\mu_i}{1-\omega_i} \end{cases}. \tag{14}$$

We can thus replace equation 14 into 13,

$$\frac{\partial \overline{W}}{\partial \overline{x}} = \int \frac{\partial(\omega_i g(x, \frac{\overline{x}-(1-\omega_i)\mu_j}{\omega_i}) + (1-\omega_i) g(x, \frac{\overline{x}-\omega_i\mu_i}{1-\omega_i}))}{\partial \overline{x}} W(x)\,dx, \tag{15}$$

which, after expanding and taking the derivatives with respect to $\overline{x}$ becomes:

$$\begin{aligned}\frac{\partial \overline{W}}{\partial \overline{x}} = &\int \omega_i \left[\frac{x - \frac{\overline{x}-(1-\omega_i)\mu_j}{\omega_i}}{\omega_i \sigma^2}\right] g(x, \frac{\overline{x}-(1-\omega_i)\mu_j}{\omega_i}) W(x)\, dx \\ &+ \int (1-\omega_i) \left[\frac{x - \frac{\overline{x}-\omega_i\mu_i}{1-\omega_i}}{(1-\omega_i)\sigma^2}\right] g(x, \frac{\overline{x}-\omega_i\mu_i}{1-\omega_i}) W(x)\, dx.\end{aligned} \tag{16}$$

To simplify notation, at this point we can come back to the original variables μ_i and μ_j. We then expand and collect terms to get:

$$\begin{aligned}\frac{\partial \overline{W}}{\partial \overline{x}} = \frac{1}{\sigma^2}\Bigg[&\int x g(x,\mu_i) W(x)\, dx - \mu_i \int g(x,\mu_i) W(x)\, dx \\ &+ \int x g(x,\mu_j) W(x)\, dx - \mu_j \int g(x,\mu_j) W(x)\, dx\Bigg].\end{aligned} \tag{17}$$

Using equations 1 and 3, we can define the following quantities,

$$\overline{W}_i = \int g(x,\mu_i) W(x)\, dx \tag{18}$$

$$\overline{W}_j = \int g(x,\mu_j) W(x)\, dx \tag{19}$$

$$\overline{x}_{w,i} = \frac{1}{\overline{W}} \int x g(x,\mu_i) W(x)\, dx \tag{20}$$

$$\overline{x}_{w,j} = \frac{1}{\overline{W}} \int x g(x,\mu_j) W(x)\, dx, \tag{21}$$

then replace them into equation 17:

$$\begin{aligned}\frac{\partial \overline{W}}{\partial \overline{x}} &= \frac{1}{\sigma^2}\Big[\overline{x}_{w,i}\overline{W}_i - \mu_i \overline{W}_i + \overline{x}_{w,j}\overline{W}_j - \mu_j \overline{W}_j\Big] \\ &= \frac{1}{\sigma^2}\Big[\overline{W}_i(\overline{x}_{w,i} - \mu_i) + \overline{W}_j(\overline{x}_{w,j} - \mu_j)\Big].\end{aligned} \tag{22}$$

Using equation 5 and rearranging terms we get:

$$\overline{W}_i \Delta\mu_i + \overline{W}_j \Delta\mu_j = \sigma^2 h^2 \frac{\partial \overline{W}}{\partial \overline{x}}, \tag{23}$$

with $\overline{W} = \omega_i \overline{W}_i + (1-\omega_i)\overline{W}_j$. Equation 23 relates how changes in either μ_i or μ_j lead to changes in fitness, and vice-versa. However, it doesn't tell us anything as to how straying may lead to evolutionary change, or how that may feedback onto ecological dynamics. Because of this, we will once again use the change of variables in 14, to rewrite 23 as:

$$\overline{W}_i \Big(\frac{\Delta\overline{x} - (1-\omega_i)\Delta\mu_j}{\omega_i}\Big) + \overline{W}_j \Big(\frac{\Delta\overline{x} - \omega_i \Delta\mu_i}{1-\omega_i}\Big) = \sigma^2 h^2 \frac{\partial \overline{W}}{\partial \overline{x}}, \tag{24}$$

which can be simplified and rearranged to obtain an expression for the change in the mean phenotype of the mixture distribution,

$$\Delta\overline{x} = \Big[\sigma^2 h^2 \frac{\partial \overline{W}}{\partial \overline{x}} + \Big(\frac{(1-\omega_i)}{\omega_i}\overline{W}_i \Delta\mu_j + \frac{\omega_i}{1-\omega_i}\overline{W}_j \Delta\mu_i\Big)\Big] \frac{\omega_i(1-\omega_i)}{(1-\omega_i)\overline{W}_i + \omega_i \overline{W}_j}. \tag{25}$$

By multiplying 25 by $\frac{\overline{W}}{\overline{W}}$, we can further rewrite the equation as:

$$\Delta\overline{x} = \Big[\sigma^2 h^2 \frac{\partial \ln \overline{W}}{\partial \overline{x}} + \frac{1}{\overline{W}}\Big(\frac{(1-\omega_i)}{\omega_i}\overline{W}_i \Delta\mu_j + \frac{\omega_i}{1-\omega_i}\overline{W}_j \Delta\mu_i\Big)\Big] \frac{\omega_i(1-\omega_i)\overline{W}}{(1-\omega_i)\overline{W}_i + \omega_i \overline{W}_j}, \tag{26}$$

which has a similar form to Lande's equation (10), but shows the explicit dependence of the change in the mean of the mix on the proportion of straying individuals. A symmetrical expression can be derived for the other site.

While some understanding can be gained using equation 26, the expression does not easily allow us to simulate the change in $\overline{x}$ over time because the change in μ_i and μ_j is difficult to track in closed form. Moreover, this equation only holds for the first time step, and as new dispersing individuals arrive and the trait distribution is again updated by the influx of individuals from the other site, the mix becomes a mix of mixes, such that the analytical expressions presented above no longer hold.

The Gaussian approximation used in the main text The above issue presents us with a tradeoff: we can either track changes in phenotype exactly for a single generation, or we can track changes in phenotype across multiple generations using an approximation. Given that our central goal is to gain an understanding of the feedback between evolutionary and ecological processes in salmon populations with dispersal in explicit space, we simplify the complexities created by phenotypic evolution by assuming that, as new migrants arrive, the mixture distribution can be locally approximated by a Gaussian distribution with a mean equal to that of the mix. Based on this assumption, we can directly use Lande's equation (here equation 10), without violating the original assumptions that led to its current form.

The model used in the main text thus assumes the existence of a normally distributed trait in each population with means μ_i and μ_j. After straying occurs, the populations now have a mixture distribution as in 12. To keep track of the mean of that mix, we approximate the mixture distribution as a Gaussian with mean $\overline{x} = \omega_i \mu_i + (1 - \omega_i)\mu_j$. Selection and reproduction thus occur at the same time leading to a new generation with a normally distributed trait distribution. In a previous paper by Ronce and Kirkpatrick [3], it was shown that assumptions similar to those made here do not qualitatively alter the observed ecological or evolutionary dynamics.

[1] Falconer DS, 1967 *Introduction to Quantitative Genetics.* Edinburgh: Oliver and Boyd LTD

[2] Lande R, 1976 Natural selection and random genetic drift in phenotypic evolution. *Evolution* **30**, 314–334

[3] Ronce O, Kirkpatrick M, 2001 When sources become sinks: migrational meltdown in heterogeneous habitats. *Evolution* **55**, 1520

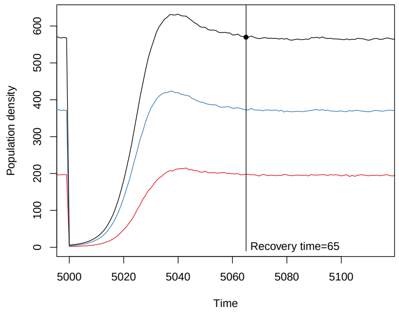

Figure 1: Example of the numerical procedure used to estimate recovery time. After a disturbance is introduced, the recovery time is calculated by measuring the point in time where N_T (in black), which is the aggregate of both populations (blue, red), settles to within one standard deviation of the new equilibrium N_T^*.

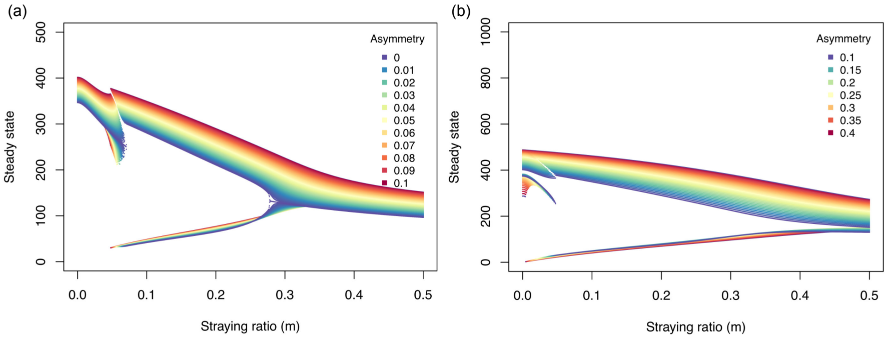

Figure 2: The effects of asymmetrical vital rates on the formation of dominant/subordinate states as a function of straying m. (a) Steady state densities for populations with symmetrical values ($\alpha = 0$) in vital rates $r_{\max}$ and β are shown with cool (blue) tones. As the asymmetry among populations between sites increases ($\alpha > 0$), vital rates diverge, such that the maximal growth at sites 1 and 2 is now $r_{\max,1} = (1+\alpha)r_{\max,2}$ and $\beta_1 = (1+\alpha)\beta_2$ where α is increased from 0 to 0.1. Steady-states for populations with increasingly asymmetric values are shown in warmer (red) tones. (b) Steady states for populations with higher asymmetric values ($0.1 \leq \alpha \leq 0.4$) for vital rates $r_{\max}$ and β. Note the change in scale compared to panel (a). If asymmetry in vital rates is very high, the pitchfork bifurcation occurs at values of $m < 0$. We observe that increasing the asymmetry in vital rates – even to very high levels – does not impact the qualitative nature of the dynamics.

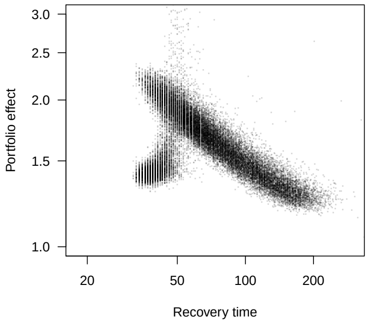

Figure 3: Comparison of portfolio effects vs. recovery time following the near-collapse of both populations where straying is assumed to be density-independent. Parameter values used are those reported in table 1, with values of $0 < h^2 \leq 0.5$ and for $0 < m \leq 0.5$.
